# Elevated gaseous luminal nitric oxide and circulating IL-8 as features of *Helicobacter pylori*-induced gastric inflammation

**DOI:** 10.48101/ujms.v126.8116

**Published:** 2021-10-15

**Authors:** Hiwa K. Saaed, Lisa Chiggiato, Dominic-Luc Webb, Ann-Sofie Rehnberg, Carlos A. Rubio, Ragnar Befrits, Per M. Hellström

**Affiliations:** a Department of Medical Sciences, Gastroenterology and Hepatology Unit, Uppsala University, Uppsala, Sweden; b Department of Gastroenterology and Hepatology, Karolinska University Hospital Solna, Karolinska Institute, Stockholm, Sweden; c Department of Pathology, Karolinska University Hospital Solna, Karolinska Institute, Stockholm, Sweden

**Keywords:** Nitric oxide, *Helicobacter pylori*, inflammation, cytokines, biomarkers

## Abstract

**Background:**

Gastric nitric oxide (NO) production in response to *Helicobacter pylori* via inducible nitric oxide synthase (iNOS) is suggested as a biomarker of inflammation and cytotoxicity. The aim of this study was to investigate relationships between gastric [NO], immunological biomarkers and histopathology.

**Materials and methods:**

Esophagogastroduodenoscopy was done in 96 dyspepsia patients. Luminal [NO] was measured by chemiluminescence. Biopsies were taken from gastric antrum and corpus for culture and histopathology. *H. pylori* IgG was detected by immunoblot assay. Biobanked plasma from 76 dyspepsia patients (11 *H. pylori* positives) was analyzed for 39 cytokines by multiplexed ELISA.

**Results:**

*H. pylori*-positive patients had higher [NO] (336 ± 26 ppb, mean ± 95% CI, *n* = 77) than *H. pylori*-negative patients (128 ± 47 ppb, *n* = 19) (*P* < 0.0001). Histopathological changes were found in 99% of *H. pylori*-positive and 37% of *H. pylori*-negative patients. Histopathological concordance was 78–100% between corpus and antrum. Correlations were found between gastric [NO] and severity of acute, but not chronic, inflammation. Plasma IL-8 (increased in *H. pylori* positives) had greatest difference between positive and negative groups, with eotaxin, MIP-1β, MCP-4, VEGF-A, and VEGF-C also higher (*P* < 0.004 to *P* < 0.032). Diagnostic odds ratios using 75% cut-off concentration were 7.53 for IL-8, 1.15 for CRP, and 2.88 for gastric NO.

**Conclusions:**

Of the parameters tested, increased gastric [NO] and circulating IL-8 align most consistently and selectively in *H. pylori*-infected patients. Severity of mucosal inflammatory changes is proportional to luminal [NO], which might be tied to IL-8 production. It is proposed that IL-8 be further investigated as a blood biomarker of treatment outcomes.

## Introduction

A troubling feature of *Helicobacter pylori* (*H. pylori*) is its persistence within the stomach via a combination of host, environmental, and bacterial factors (1, 2). Attachment of *H. pylori* to epithelial cells mediates gastric mucosal inflammation, including infiltration of neutrophils and monocytes into the gastric mucosa (3, 4). Gene and protein expressions of inducible nitric oxide (NO) synthase (iNOS, a product of NOS2 gene) are increased in gastric tissue in *H. pylori* infection (5). Simultaneous production of NO and O^2^ radicals presumably leads to nearly instantaneous combination, yielding highly reactive peroxynitrite (ONOO^–^). The extent to which NO itself can be detected and measured in real time as a feature of *H. pylori* infection *in vivo* is, therefore, unclear. Previous studies exploring cytokine panels of blood protein and tissue RNA concentration demonstrated the production of many immune signaling molecules in *H. pylori* infection (6, 7), albeit with somewhat different outcomes. For example, interleukins (IL)-6 and IL-8 are considered primary signals in infection but did not reach significance compared to controls across both these studies. IL-6 is thought to drive C-reactive protein (CRP) production, which is routinely measured in clinical chemistry. However, CRP has been shown to not be associated with *H. pylori* infection (8). Further investigations are needed to build concensus on human *in vivo* pathophysiology in *H. pylori* infection as to whether NO is detectable in the gastric lumen, whether detected NO is traceable to iNOS, which circulating cytokines are most likely tied to NO and *H. pylori* under different conditions (e.g. acute vs chronic inflammation), and also how cytokines relate to CRP.

The aim of this study was to assess relationships between *H. pylori* infection and direct measurements of gastric luminal gaseous NO concentration [NO] with inflammatory-associated immunological and histopathological findings. Separately, a 39-plex inflammatory biomarker panel was used to determine which plasma markers were most significantly associated with *H. pylori* infection. Correlation analysis was performed across all plasma markers.

## Materials and methods

### Study subjects

In a first study group, 96 adult patients (56 women and 40 men, mean age 39, range 17–86 years) referred for routine diagnostic upper gastrointestinal endoscopy, were included for NO measurement and biopsy investigations. Inclusion criteria were age 17–90 years with dyspeptic symptoms, either as postprandial distress or epigastric pain syndrome. Exclusion criteria were peptic ulcer disease, history of gastric surgery, use of proton pump inhibitors or non-steroidal antiinflammatory drugs, and ongoing upper gastrointestinal bleeding or delayed gastric emptying (defined as remaining gastric contents at endoscopy after a 4-h fasting period). In a second study group, inflammatory biomarker analysis was carried out on plasma samples obtained from a local biobank of patients referred for dyspepsia and upper gastrointestinal symptoms (11 *H. pylori*-positive and 65 *H. pylori*-negative patients; 46 women and 30 men, mean age 54, range 19–84 years). The same inclusion and exclusion criteria were applied to both groups.

### Endoscopy clinical protocol

Patients were fasted overnight. Endoscopy was then carried out between 8:00 and 11:00 am the following day. All patients were offered surface anesthesia of the gullet by lidocaine (Xylocaine spray; 10 mg/mL, Astra Zeneca, Södertälje, Sweden) and intravenous midazolam administered at a dose of 0.06 mg/kg (1 mg/mL, Algol Pharma, Espoo, Finland).

### Nitric oxide measurements

Luminal [NO] was measured as the initial step of each endoscopy session. Gastric air (50 mL) was withdrawn using a 60 mL syringe with a suction catheter through the working channel of the endoscope. The sampled air was immediately injected into a chemiluminescence analyzer (CLD 700, Eco Physics, Dürnten, Switzerland) for the analysis of the peak gaseous [NO]. The chemiluminescence method for gas analysis of [NO] relies on the measurement of light produced by the gas-phase titration of NO and ozone. NO is unstable, oxidizing to NO^2^ in the presence of ozone. This reaction produces a quantity of light for each NO molecule oxidized, which is detected by a photomultiplier tube. Because volumes of sample gas and excess ozone are controlled, light intensity in the reaction chamber is proportional to [NO]. Lower detection limit of [NO] was one part per billion (ppb). Analyzer calibration was done routinely using nitrogen with NO at known concentrations spanning 10–10,000 ppb (AGA AB, Lidingö, Sweden).

### Microbiology

At endoscopy, biopsies were taken for culture, one from gastric antrum and one from corpus. Blood was drawn and plasma prepared for use in serological diagnosis as previously described (9–11). *H. pylori* IgG antibodies were detected by Western blot (Helicoblot 2.1, MP Biomedicals Germany GmbH, Eschwege, Germany) according to manufacturers’ instructions.

### Histopathology

At endoscopy, two biopsies were taken from antrum and two from corpus that were placed in separate vials containing 4% buffered formalin. Hematoxylin–eosin and Giemsa staining were performed. The presence of the parameters acute inflammation, chronic inflammation, *H. pylori*, mucosal atrophy, intestinal metaplasia, and pseudopyloric metaplasia was individually classified into grade 1 (with one or more foci having one on the above parameters) or grade 2 (where a parameter was present across the entire biopsy section) as reported previously (12). Grading was made by two observers (CAR and ASR) blinded to *H. pylori* status and [NO], as well as to morphological findings during endoscopy. Grading of each subject’s set of biopsies was used to calculate histopathological scores in biopsy pairs from antrum and corpus. Histopathological scores were calculated by summation of the grades of each biopsy, multiplied by number of biopsies displaying that specific finding. Maximum score was 8.

### Classification of patients

*H. pylori* status of each patient was assessed individually and classified based on culture, immunoblot, and histopathology. Confirmation by culture was in all subjects scored as positive *H. pylori* status.

### Cytokines and chemokines

Using the 76 biobanked plasma samples (46 women and 30 men, mean age 54, range 19–84 years), *H. pylori*-positive patients (*n* = 11) were compared with *H. pylori*-negative patients (*n* = 65). Chemokines and cytokines were quantified using a human cytokine 39-plex ELISA kit (Meso Scale Diagnostics, Rockville, MD, USA). Analytes were as follows: CRP, basic fibroblast growth factor (bFGF), eotaxin, eotaxin-3, Fms-related receptor tyrosine kinase 1 (Flt-1), granulocyte-macrophage colony-stimulating factor (GM-CSF), intercellular adhesion molecule 1 (ICAM-1), interferon (INF)-g, interferon inducible protein (IP)-10, IL-1a, IL-1b, IL-2, IL-4, IL-5, IL-6, IL-7, IL-8, IL-10, IL-12p70, IL-12/IL-23p40, IL-13, IL-15, IL-16, IL-17A, macrophage-derived chemokine (MDC), macrophage inflammatory protein (MIP)-1a, MIP-1b, monocyte chemotactic protein (MCP)-1, MCP-4, placental growth factor (PIGF), serum amyloid A (SAA), thymus activation-regulated chemokine (TARC), tumor necrosis factor (TNF)-a, TNF-b, tyrosine-protein kinase receptor (Tie-2), vascular cell adhesion protein 1 (VCAM-1), vascular endothelial growth factor (VEGF-A), VEGF-C, and VEGF-D. Samples were analyzed in duplicate with electrochemiluminescence.

### Statistical analysis

Statistical analyses for significant differences in biochemical variables between *H. pylori*-positive and *H. pylori*-negative patients were performed using Student’s *t*-test or Mann–Whitney U-test for data that were not normally distributed. For histopathology, comparisons were made with the Mann–Whitney U-test. Analysis for simple linear parametric correlations used Pearson’s method (*P*  < 0.05 was deemed significant). Data were similarly assessed by Spearman’s rank method to account for outliers and non-parametric or curvilinear correlations. Diagnostic odds ratio (DOR) was calculated using the 75% percentile cut-off according to the formula DOR = (true positive/false negative)/(false positive/true negative). Histopathological results were regarded as gold standard (13).

### Ethics

This study was approved by the Ethics Committee North at Karolinska University Hospital, Solna (Dnr 97-034; 1997-06-18 and 99-119; 1999-11-24). Verbal and written informed consents were obtained before performing endoscopy. The study protocol conforms to the ethical guidelines of the 1975 Declaration of Helsinki as reflected in *a priori* approval by the institution’s human research committee.

## Results

### Endoscopic findings

In the group of dyspeptic patients undergoing endoscopy, 77 were *H. pylori*-positive patients (mean age 37, range 17–61 years), whereas 19 were *H. pylori*-negative patients (mean age 39, range 34–86 years). All patients had a similar clinical picture of functional dyspepsia with either epigastric pain syndrome or postprandial fullness syndrome.

**Figure. 1:**
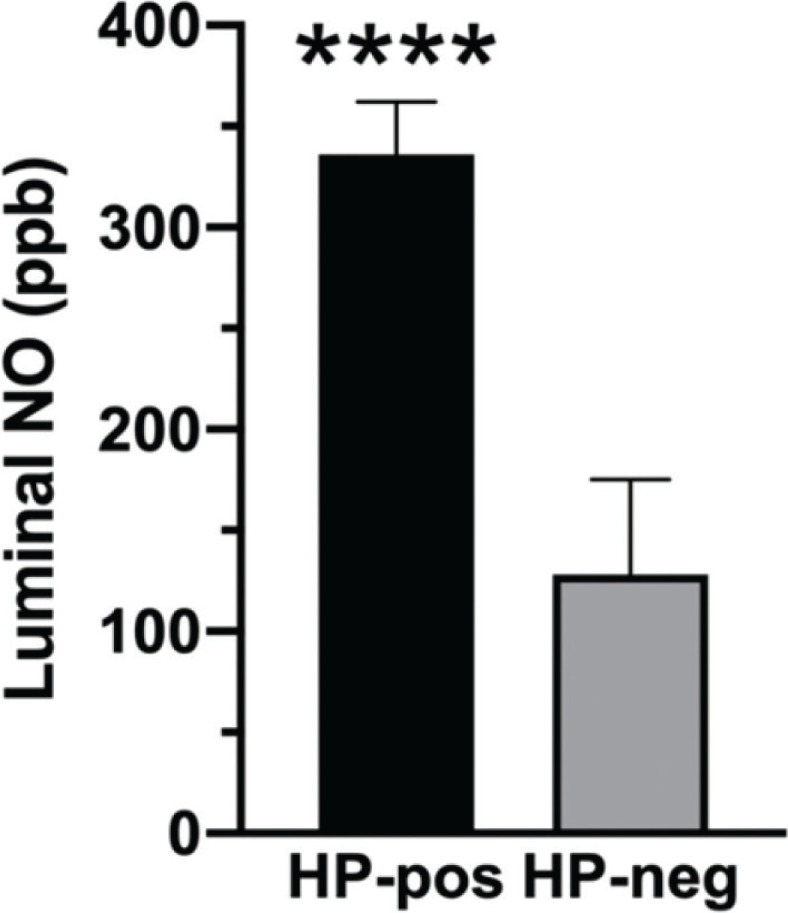
Luminal [NO] in the stomach of *Helicobacter*-positive (HP-pos, *n* = 77) as compared with *Helicobacter*-negative (HP-neg, *n* = 19) patients. ****P < 0.0001. Data are mean ± SEM.

*H. pylori*-positive patients had higher gastric luminal [NO] than *H. pylori*-negative subjects (336 ± 26 vs 128 ± 47 ppb; *P* < 0.0001) (Figure 1).

Among *H. pylori*-positive patients, 76 out of 77 were found to have histopathological changes of the mucosa, whereas in the *H. pylori*-negative control group, only 7 of 19 patients had histopathological changes. Concordance in histopathology between paired biopsies from corpus and antrum was high; 78–100% of the paired biopsies received the same histological grade. In gastric antrum, histopathological changes of *H. pylori*-infected patients showed not only acute and chronic inflammations (*P* <0.004) with infiltration of lymphocytes and plasma cells but also structural changes with mucosal atrophy and metaplasia approaching significance (*P* < 0.087), as compared to non-infected antrum. Mucosal inflammatory and atrophic changes showed stratefication at scoring 2 with moderate inflammation. In corpus, histopathology showed inflammation (*P* < 0.001) as well as structural changes with mucosal atrophy and metaplasia (*P* < 0.029). Again, acute inflammatory and atrophic changes were moderate with stratefication at scoring 2, whereas chronic inflammation was more severe and scored 4 (Table 1).

**Table 1:** Histopathological scoring of the gastric antrum (top) and corpus (bottom) in *Helicobacter*-positive and *Helicobacter*-negative patients.

Antrum	Acute inflammation		Chronic inflammation		Mucosal atrophy		Intestinal metaplasia		Pseudopyloral metaplasia	
Score	*H. pylori*		*H. pylori*		*H. pylori*		*H. pylori*		*H. pylori*	
	+ve	−ve	+ve	−ve	+ve	−ve	+ve	−ve	+ve	−ve
0	24	18	12	15	52	16	71	18	71	17
1	5	0	3	2	8	1	2	0	2	0
2	44	1	37	1	15	1	4	1	4	2
3	0	0	7	0	0	0	0	0	0	0
4	4	0	18	1	2	1	0	0	0	0

**Total**	**77**	**19**	**77**	**19**	**77**	**19**	**77**	**19**	**77**	**19**

**Table:** 

Corpus	Acute inflammation		Chronic inflammation		Mucosal atrophy		Intestinal metaplasia	
Score	*H. pylori*		*H. pylori*		*H. pylori*		*H. pylori*	
	+ve	−ve	+ve	−ve	+ve	−ve	+ve	−ve
0	9	18	3	16	28	17	65	18
1	8	0	1	0	13	0	5	0
2	55	1	24	3	33	2	6	0
3	1	0	9	0	2	0	1	0
4	4	0	40	0	1	4	0	1

**Total**	**77**	**19**	**77**	**19**	**77**	**19**	**77**	**19**

*H. pylori*: *Helicobacter pylori*; +ve: positive; −ve: negative.

**Figure. 2:**
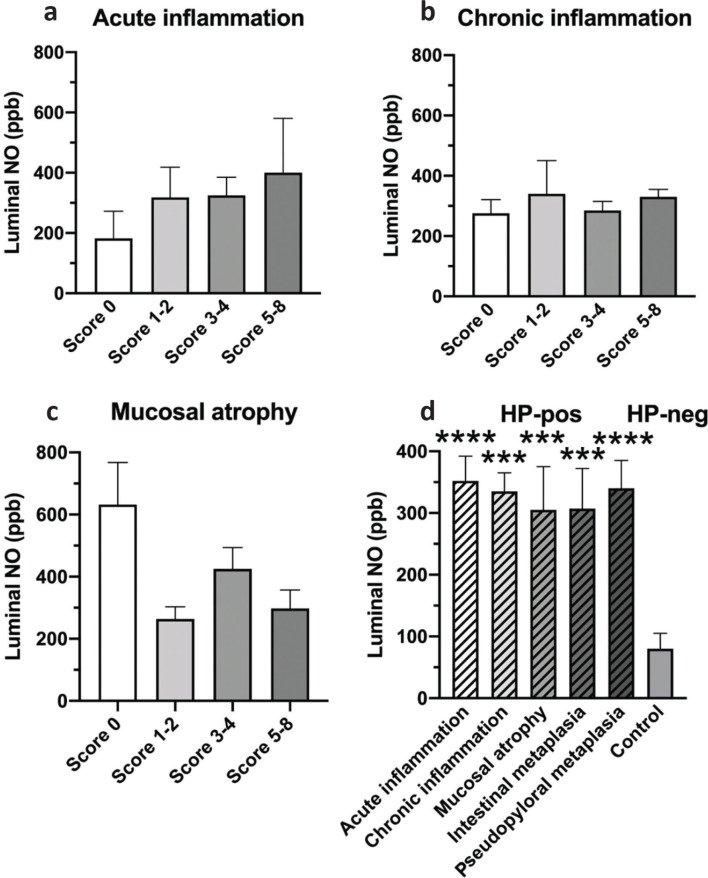
Luminal [NO] in antrum in relation to histopathology scores. (a) Luminal NO concentration relative acute inflammation. Number of patients were [score(*n*)]: 0(5); 1–2(25); 3–4(40); 5–8(7). (b) Luminal [NO] relative chronic inflammation. Number of patients were [score(*n*)]: 0(3); 1–2(4); 3–4(25); 5–8(45). (c) Luminal [NO] relative mucosal atrophy. Number of patients were [score(*n*)]: 0(23); 1–2(35); 3–4(16); 5–8(3). Scores in A, B, and C are for *H. pylori*-positive group only. Data are mean ± SEM. (d) Histopathology by positive clinical finding independent of score. In histopathology, more than one positive finding can occur in the same visual field. Data are mean ± SEM, left to right, *n* = 36, 67, 12, 14, 15, and 12). Control is *H. pylori-negative* by histopathology. ***P < 0.001, ****P < 0.0001.

Figure 2 shows [NO] relative histopathology scores. In *H. pylori* positives, a correlation was found between gastric luminal [NO] and acute inflammation scores (Figure 2a). This trend was not observed in scores of chronic histopathological changes (Figure 2b). Conversely, luminal [NO] was lower in the presence of mucosal atrophy (Figure 2c). In relative controls, [NO] was elevated to a similar extent in those with the presence of acute or chronic inflammation, mucosal atrophy, and intestinal or pseudopyloral metaplasia (Figure 2d).

### Blood sample findings

In the study group from which biobanked plasma was analyzed for circulating cytokines and chemokines, IL-8 was elevated the most in *H. pylori*-positive patients, with eotaxin, MIP-1β, MCP-4, VEGF-A, and VEGF-C also higher (*P* < 0.004 to *P* < 0.032) (Table 2). DOR values using the 75% percentile cut-off for true positives were 7.53 for IL-8 as compared with 1.15 for CRP and 2.88 for gastric [NO].

**Table 2:** Cytokine and chemokine plasma concentrations in *Helicobacter*-positive (*n* = 11) and *Helicobacter*-negative (*n* = 65) subjects. Data are median ± SD. Statistics are Mann–Whitney ranked sum test. Units are ng/L (see footnotes for exceptions). Significant differences are in bold.

Cytokine/chemokine	Lower limit of quantification (LLOQ)^2^	Helicobacter pylori		*P*
		Positive	Negative	
CRP^1^	27.6	1.95 ±9.72	1.01 ±5.85	0.36
bFGF	2.60	3.27 ±4.26	2.29 ±12.72	0.44
**Eotaxin**	12.3	**92.81 ± 52.11**	**70.40 ± 43.02**	**0.022**
Eotaxin-3	10.2	14.72 ±8.5	16.23 ±23.66	0.39
Flt-1	10.0	41.66 ±9.11	36.29 ±12.46	0.44
GM-CSF	0.842	0.15 ±0.10	0.29 ±0.35	0.11
ICAM-1^1^	15.0	0.22 ±0.08	0.22 ±0.11	0.84
IFN-γ	1.76	3.44 ±3.05	3.98 ±4.54	0.50
IP-10	1.37	213.6 ±962.74	182.10 ±367.3	0.79
IL-1α	2.85	4.67 ±4.25	4.77 ±6.22	0.72
IL-1β^3^	0.646	0.08 ±0.05	0.11 ±0.59	0.75
IL-2^3^	0.890	0.28 ±0.18	0.17 ±0.58	0.53
IL-4	0.218	0.08 ±0.07	0.06 ±0.46	0.74
IL-5	4.41	0.66 ±0.64	0.58 ±0.55	0.85
IL-6	0.633	0.46 ±1.03	0.49 ±0.65	0.71
IL-7	0.546	5.7 ±6.52	2.11 ±2.08	0.08
**IL-8**	0.591	**5.87 ±2.11**	**2.52 ±3.08**	**0.004**
IL-10	0.298	0.34 ±0.30	0.30 ±0.83	0.91
IL-12p70	1.22	0.30 ±16.4	0.27 ±0.70	0.74
IL-12/IL-23p40	1.32	87.41 ±54.51	94.22 ±80.78	0.99
IL-13	4.21	0.94 ±0.52	1.09 ±2.01	0.66
IL-15	0.774	1.20 ±0.44	1.25 ±0.63	0.68
IL-16	19.1	116.28 ±23.59	108.70 ±51.75	0.51
IL-17A	3.19	4.77 ±2.65	4.28 ±4.53	0.99
MDC	88.3	829.31±360.99	559.42 ±290.62	0.12
MIP-1α	13.8	19.95 ±6.93	16.15 ±7.74	0.11
**MIP-1β**	1.02	**94.63 ±38.93**	**63.85 ±105.08**	**0.014**
MCP-1	1.09	61.99 ±107.01	39.65± 48.06	0.33
**MCP-4**	5.13	**81.84 ±93.43**	**56.60 ±42.09**	**0.023**
PIGF	1.50	5.04 ±1.04	4.42 ±1.94	0.38
SAA^1^	54.0	2.22 ±26.67	2.61 ±16.36	0.55
TARC	3.32	45.69 ±167.40	31.85 ±102.53	0.42
TNF-α	0.690	1.87 ±0.67	1.67 ±1.08	0.65
TNF-β	0.465	0.42 ±0.11	0.47 ±0.34	0.28
Tie-2	396	850.89 ±169.84	816.00 ±219.69	0.43
VCAM-1^1^	37.6	0.22 ±0.10	0.28±0.14	0.30
**VEGF-A**	5.00	**29.33±149.01**	**18.68±42.32**	**0.027**
**VEGF-C**	146	**110.53 ±500.44**	**56.10 ±230.04**	**0.032**
VEGF-D	67.1	2167.99 ±1289.84	1833.37 ±718.70	0.24

CRP: C-reactive protein; bFGF: basic fibroblast growth factor; GM-CSF: granulocyte-macrophage colony-stimulating factor; ICAM-1: intercellular adhesion molecule 1; IP, interferon inducible protein; IFN-g: interferon-gamma; IL, interleukins; Flt-1, Fms-related receptor tyrosine kinase 1; MDC: macrophage-derived chemokine; MIP: macrophage inflammatory protein; MCP: monocyte chemotactic protein; PIGF: placental growth factor; SAA: serum amyloid A; TARC: thymus activation-regulated chemokine; TNF: tumor necrosis factor; VCAM-1: vascular cell adhesion protein 1; VEGF: vascular endothelial growth factor.

1 Plasma concentrations (but not LLOQ [lower limit of quantification]) are mg/L due to their comparatively high concentrations.

2 LLOQ values are taken from product certificate of analysis and were consistent with calculations from experimental results. Plasma concentrations below LLOQ (e.g. VEGF-C) were above lower limit of detection, but must be considered unreliable.

3 Many samples were below detection limit, in which case no value was obtained.

Correlation analysis for plasma cytokines was done with combined data from *H. pylori* positives and negatives. For 39 analytes, there are 741 comparison pairs per test. Pearson’s and Spearman’s methods gave similar results. Complete data from Pearson’s method are available in Supplementary Table 1. Numerous significant correlations were found. Terms like ‘moderate’ or ‘strong’ are arbitrary. By setting a lower cutoff for moderate correlations as R > 0.50 or <−0.50 (i.e. antiparallel) as well as *P* <0.05, Pearson’s test identified 45 comparisons meeting this criteria, whereas Spearman’s test identified 42. There was substantial overlap in which same analyte pairs correlated. By setting a cutoff for strong correlations as R > 0.66 or < −0.66 (i.e. antiparallel) as well as *P* < 0.001, Pearson’s test identified 19 comparisons meeting this criteria, whereas Spearman’s test identified 6. Importantly, IL-8 did not correlate even by the moderate criteria with any other analyte in either test. Hence, by the criteria above, IL-8 was an independent measure. For comparison, IL-6 (believed to drive acute phase liver proteins) had consistent moderate or strong correlations with CRP and SAA across both tests. In both tests, IL-8 vs CRP correlation was close to 0, with *P* ~ 0.5 (i.e. no correlation). It can, therefore, be argued that IL-8 is a more reliable indicator of *H. pylori* infection than CRP. Since samples were stored many years in a biobank, it is likely that IL-8 is also sufficiently stable for use as biomarker of infection.

## Discussion

Many researchers have assumed increased NO elaboration based on the expression of different NOS isoforms (14, 15). Higher gastric [NO] was, indeed, found in *Helicobacter*-infected subjects.

NO has a dual role: constitutive at low concentrations for maintaining physiological functions (16) and inducible during inflammatory reactions, even reaching cytotoxic concentrations (17). Basal mucosal NO production originates from nNOS and eNOS present in the gastric mucosa. Higher concentrations result from upregulated iNOS in gastric epithelial cells (18) as well as neutrophils and macrophages (19). In support of this, luminal [NO] measurement in the gastrointestinal tract has proven useful in inflammatory bowel disease, infective gastroenteritis, and celiac sprue, where similar inflammatory mechanisms are thought to occur (20–22).

Elevated [NO] is generated by upregulated iNOS triggered by inflammation or tissue injury in gastric epithelial cells (15, 18, 23). This has also been reported for neutrophils and macrophages exposed to *H. pylori* (19), consistent with NO as an acute proinflammatory agent (24). The inducibility of iNOS seems, however, to be reversible. Increased iNOS expression in *H. pylori*-associated chronic gastritis diminishes and returns to baseline once *H. pylori* is eradicated (17).

Some researchers have found lower luminal [NO] using a similar chemiluminescence measurement technique (25–27). Differences in results may be due to difficulties in the sampling techniques with dilution of gaseous samples. In the present study, luminal suctions were carried out in a standardized manner but still may involve a certain degree of aerial dilution. Swallowed air during the intubation procedure may have further diluted the NO samples. Taken together, these two factors may have mitigated the [NO] issues in this study. The *H. pylori* infection in publications of Fändriks et al. (25) and Lundberg et al. (26) may have reached a broader state of chronicity and mucosal atrophy with extended chronic gastritis being less capable of producing NO, but still harboring *H. pylori.* However, the normal distribution of the NO data, together with the marked differences between *H. pylori*-positive and *H. pylori*-negative patients speaks in favor of the NO measurements being representative of an inflammatory disease process.

Other authors have also found higher gastric [NO] in *H. pylori*-infected than non-infected subjects with an associated prognosis of chronic gastritis and atrophy of the corpus. The latter being known to increase gastric pH (15), but with no histopathological investigation to confirm this. Likewise, Shiotani et al. (12) found elevated pH and increased nitrites in gastric juice during *H. pylori*-infection. This correlated with degree and spread of gastritis and atrophy. Otherwise, Shiotani et al. (27) in another study reported low gastric [NO] with increased nitrites in gastric juice as a result of an elevated pH in gastric juice in patients with long-standing *Helicobacter* infection (27).

The association between the presence of *H. pylori* and continuous disease process is complicated. A disconnection between the rate of NO synthesis and augmented iNOS expression has been described with a similar NO formation in *H. pylori* infected and non-infected antral mucosa despite elevated iNOS activity in the *H. pylori* infected. This finding was explained by unique ability of *H. pylori* to produce a competitive NOS inhibitor down-regulating the L-arginine/NO-pathway to evade host defense mechanisms (15). Gobert *et al*. reported that *H. pylori* expresses the gene rocF that encodes arginase, which depletes arginine, effectively dampening NO production by iNOS in macrophages (28).

Positive trends were found between [NO] and the score of acute inflammation in the mucosa of antrum and corpus, as well as to serum concentrations of anti-*H. pylori* IgG, but with no statistical significance. This might be due to the successive development of an atrophic mucosa not capable of producing NO. Another reason for these results being disparate from two other human studies (25, 29) could be the younger age of our present patients. The age factor might well influence the results due to the paucity of the development of gastric atrophy in young compared to old individuals. This is supported by the histopathological findings in our patients, in which chronic gastritis in antrum was more frequent than in corpus in younger patients. This implies that acid secretion was still normal, and intragastric reduction of nitrite/nitrate to NO was preserved. This is also in agreement with data showing a trend toward lower [NO] among *H. pylori*-infected patients with mucosal atrophy (12).

*H. pylori* infection has been reported to induce proinflammatory cytokines and chemokines, such as IL-1β, IL-6, IL-8, IL-23, and TNF-α, where the IL-8 secreted from gastric mucosal epithelium was the major chemokine driving inflammatory response (30). In the present study, IL-8 had strongest significance (*P* = 0.004) and largest fold difference (2.8-fold higher in *H. pylori* positives) among analytes. IL-8 also had the highest DOR, including by comparison to NO or CRP. It is possible for iNOS and IL-8 to coordinate acute gastric inflammation in a common signaling pathway. Released NO can activate oxidant sensitive transcription factors. NO donors can induce increased concentrations of IL-8 and nitrite as well as a time-dependent mRNA expression of IL-8 in cell lines, indicating signaling between the l-arginase/NO pathway and IL-8 that is activated by *Helicobacter* (31)*.*

Elevated eotaxin suggests an eosinophilic component of *H. pylori* inflammatory response. Eosinophils may cross the endothelium to penetrate into gastric inflammatory tissue by a regulated process through a coordinated interaction between networks involving chemokine eotaxin-1, eosinophil adhesion molecules, and adhesion receptors on the endothelium (32). Elevated plasma MCP-4 concentrations in *H. pylori*-positive patients are consistent with its production at the infection site. MCP-4 induction has been identified in complex tissue proteomes (33). Furthermore, MIP-1β has previously been shown to be upregulated in *Helicobacter*-infected monocytes (34), which, together with IL-8, may be associated with neutrophil infiltration characteristic of *H. pylori*-infected mucosa (35). To this end, VEGF concentrations, primarily of the A and C subtypes considered to be endothelial cell specific, were elevated. Together with IL-8, VEGF has previously been found to be increased in gastric epithelial cells during *H. pylori* infection, being important for vascular remodeling in gastric epithelial cells (36). VEGF has been suggested to enhance healing of gastric injury in an iNOS-dependent manner independently of stimulated angiogenesis (37). In addition to stimulating angiogenesis to promote restoration of connective tissue and microvessels in injured mucosa, effects through iNOS downregulation exert a therapeutic effect on mucosal lesions.

Although CRP, a clinical marker of acute inflammation, averaged about double that of the control group, significance was not reached due to skewed spread in CRP concentrations. However, strong correlations between IL-6 and CRP were found, consistent with the concept that IL-6 drives CRP production. This correlation was found with this panel in Crohn’s disease patients (38). *H. pylori* infection did not consistently elevate IL-6, whereas IL-8 apparently cannot replace IL-6 to drive CRP production.

In the short term, elevated [NO] in *H. pylori*-infected patients is beneficial for the host. This benefit may be reduced with more profound tissue injury. l-Arginine-dependent NO production in neutrophils and macrophages increases when cocultured with *H. pylori* (19, 39), consistent with the present results. However, chronically elevated NO in chronic gastritis (Figure 2, panels b and d) might contribute to gastric adenocarcinoma, as by immunosuppressive nitrotyrosine production in cytotoxic T cells (40).

In conclusion, the results showed significant increases of gastric [NO] and circulating IL-8 in *H. pylori*-infected patients compared with those without *H. pylori*. Severity of mucosal inflammatory changes, however, is not unambiguously revealed by luminal [NO] alone, thus requiring histopathological assessment.

## Strengths, limitations, and clinical significance

This exploratory study had limited sample size. Age, BMI, smoking, alcohol consumpton, and other data that could be relevant for cytokine concentrations were unavailable. NO data were obtained from different patients than IL-8, VEGF, CRP, and other circulating inflammatory biomarkers; correlation analyses between NO and IL-8 were not possible. IL-8 can easily be measured with good accuracy, which is clinically important. Successful *H. pylori* treatment requires not only eradication of *H. pylori* but would also escape opportunistic secondary infections, many of which elevate IL-8. This study indicates that IL-8 may have clinical chemistry utility in *H. pylori* treatment follow-ups and outcomes.

## Supplementary Material

Elevated gaseous luminal nitric oxide and circulating IL-8 as features of Helicobacter pylori-induced gastric inflammation
