# Supplementary material for: Elevated gaseous luminal nitric oxide and circulating IL-8 as features of Helicobacter pylori-induced gastric inflammation
Source: Ups J Med Sci. 2021 Oct 15;126:10.48101/ujms.v126.8116. doi: 10.48101/ujms.v126.8116 (PMC8559587; doi:10.48101/ujms.v126.8116)
Supplement: Elevated gaseous luminal nitric oxide and circulating IL-8 as features of Helicobacter pylori-induced gastric inflammation [file UJMS-126-8116-s001.pdf]

Bold indicates R > 0.5 or < -0.5 (i.e., antiparallel) as well as P <0.05, suggesting a moderate correlation that met statistical significance.

Pearson Correlation of HP negative group
Upper number is Correlation Coefficient. Lower number is P-value

	ICAM-1	SAA	VCAM	FLT-1	PIGF	Tie-2	VEGF-C	VEGF-D	bFGF
CRP	0.021	**0.97**	0.0391	0.252	0.366	0.219	-0.181	0.708	0.0556
	0.951	**8.22E-07**	0.909	0.454	0.269	0.518	0.595	0.0147	0.871
ICAM-1		0.0704	0.594	0.248	0.579	0.52	-0.0341	-0.0197	-0.189
		0.837	0.0541	0.461	0.0621	0.101	0.921	0.954	0.578
SAA			0.133	0.177	0.382	0.243	-0.214	**0.753**	0.003
			0.697	0.603	0.246	0.472	0.527	**0.00743**	0.993
VCAM				**0.618**	0.2	0.443	-0.0119	-0.0963	-0.277
				**0.0429**	0.556	0.172	0.972	0.778	0.409
FLT-1					0.112	0.501	-0.152	-0.272	-0.0906
					0.744	0.116	0.657	0.418	0.791
PIGF						0.0664	0.0231	0.472	0.281
						0.846	0.946	0.143	0.402
Tie-2							-0.257	-0.195	-0.413
							0.446	0.566	0.207
VEGF-C								-0.201	**0.643**
								0.553	**0.0328**

VEGF-D									0.123
									0.719

	Eotaxin	eotaxin-3	IP-10	MCP-1	MCP-4	MDC	MIP-1a	MIP-1b	TARC
CRP	-0.548	-0.661	-0.165	-0.282	-0.117	-0.121	0.328	**-0.877**	-0.273
	0.16	0.0527	0.628	0.4	0.782	0.739	0.428	**0.0019**	0.446
ICAM-1	0.0127	0.322	-0.0695	-0.221	0.233	-0.124	0.401	-0.127	0.193
	0.976	0.398	0.839	0.515	0.578	0.732	0.325	0.745	0.593
SAA	-0.443	-0.653	-0.198	-0.275	-0.176	0.0163	0.314	**-0.847**	-0.281
	0.272	0.0567	0.56	0.413	0.677	0.964	0.448	**0.00393**	0.432
VCAM	-0.379	0.494	0.116	0.0376	-0.0462	0.345	**0.713**	0.192	0.0128
	0.354	0.176	0.734	0.913	0.913	0.328	**0.0471**	0.62	0.972
FLT-1	-0.244	0.308	0.178	-0.219	0.017	0.0612	**0.754**	-<br>0.00235	-0.16
	0.56	0.42	0.6	0.519	0.968	0.867	**0.0308**	0.995	0.658
PIGF	0.351	-0.133	0.127	-0.364	0.574	0.0775	0.298	-0.287	0.3
	0.394	0.734	0.71	0.27	0.137	0.832	0.473	0.454	0.4
Tie-2	-0.0562	0.0734	-0.0829	-0.183	-0.152	-0.282	0.522	-0.285	-0.191
	0.895	0.851	0.808	0.59	0.719	0.43	0.185	0.457	0.596

VEGF-C	0.423	0.231	**0.812**	**0.794**	**0.808**	0.359	0.0973	0.336	**0.928**
	0.296	0.55	**0.00239**	**0.00353**	**0.0152**	0.308	0.819	0.376	**0.000109**
VEGF-D	0.292	-0.526	-0.349	-0.347	-0.0854	0.201	-0.425	**-0.7**	-0.173
	0.483	0.146	0.293	0.296	0.841	0.578	0.294	**0.036**	0.633
bFGF	**0.77**	0.21	0.545	0.126	**0.94**	0.00232	0.253	0.103	**0.736**
	**0.0254**	0.587	0.0832	0.712	**0.000526**	0.995	0.546	0.792	**0.0153**
Eotaxin		0.434	0.254	-0.0866	0.641	-0.402	0.108	0.0392	0.636
		0.283	0.544	0.838	0.0866	0.323	0.8	0.927	0.0902
eotaxin-3			0.245	0.0544	0.315	0.131	0.663	**0.703**	0.363
			0.524	0.889	0.447	0.757	0.0733	**0.0348**	0.337
IP-10				**0.643**	**0.807**	0.257	0.485	0.512	**0.838**
				**0.0329**	**0.0154**	0.473	0.223	0.159	**0.00248**
MCP-1					0.307	0.535	-0.138	0.408	0.593
					0.459	0.111	0.744	0.276	0.0705
MCP-4						-0.0316	0.403	0.421	**0.964**
						0.941	0.323	0.299	**0.000112**
MDC							-0.109	0.242	0.274
							0.798	0.564	0.476
MIP-1a								0.541	0.265

								0.166	0.525
MIP-1b									0.434
									0.244
	**GM-CSF**	**IL12-23**	**IL15**	**IL16**	**IL17A**	**IL1a**	**IL5**	**IL7**	**TNFb**
CRP	0.128	0.79	0.819	0.265	0.811	0.0575	0.894	-0.0512	-0.201
	0.742	0.0038	0.00202	0.431	0.00243	**0.867**	0.00277	0.881	0.554
	0.902	0.565	0.742	0.175	0.634	**0.0291**	0.88	0.369	0.612
VCAM	-0.19	0.425	-0.0878	0.234	-0.137	0.351	0.216	-0.192	0.0622
FLT-1	0.416	0.535	0.089	-0.331	0.121	0.428	0.606	-0.173	0.0588
	0.265	0.0901	0.795	0.321	0.723	0.189	0.111	0.611	0.864
	0.689	0.126	0.182	0.274	0.358	0.161	0.509	0.531	0.973
VEGF-C	-0.296	0.136	0.244	0.106	0.0268	-0.407	0.0249	0.882	0.867
VEGF-D	-0.263	0.457	0.514	0.363	0.747	-0.176	0.573	-0.222	-0.34
	0.495	0.157	0.106	0.273	0.00823	0.605	0.137	0.512	0.306
	0.942	0.607	0.444	0.747	0.16	0.145	0.696	0.11	0.169
eotaxin-3	-0.116	-0.294	-0.634	-0.358	-0.325	-0.0367	-0.581	-0.0327	0.242
IP-10	-0.244	0.335	0.265	-0.03	-0.0366	-0.265	0.0633	**0.807**	**0.887**
	0.527	0.313	0.43	0.93	0.915	0.431	0.882	**0.0027**	**0.000275**

MCP-4	-0.131	0.516	0.702	0.126	0.53	-0.233	0.38	0.621	0.774
	0.779	0.19	0.0523	0.767	0.177	0.579	0.528	0.1	0.0241
MDC	-0.656	0.282	-0.176	0.0937	-0.139	0.0142	0.282	0.183	0.567
	0.0775	0.43	0.627	0.797	0.702	0.969	0.541	0.613	0.0877
MIP-1a	0.00104	**0.833**	0.0992	0.0258	0.191	0.121	0.66	0.0636	0.29
	0.998	**0.0102**	0.815	0.952	0.651	0.775	0.225	0.881	0.485
MIP-1b	-0.31	-0.454	**-0.674**	-0.407	-0.659	-0.192	**-0.818**	0.22	0.499
	0.456	0.22	**0.0466**	0.276	0.0534	0.621	**0.0466**	0.57	0.171
TARC	-0.385	0.115	0.201	0.185	-0.00204	-0.288	-0.105	**0.75**	**0.871**
	0.307	0.752	0.578	0.609	0.996	0.421	0.823	**0.0125**	**0.00104**
GM-CSF		-0.0534	-0.0258	**-0.699**	0.0651	0.226	-0.0844	-0.293	-0.437
		0.892	0.948	**0.036**	0.868	0.558	0.874	0.444	0.24
IL12-23			**0.787**	0.21	**0.665**	0.0539	**0.813**	0.181	0.186
			**0.00403**	0.536	**0.0254**	0.875	**0.0142**	0.594	0.584
IL15				0.357	**0.705**	-0.0556	**0.857**	0.385	0.163
				0.281	**0.0154**	0.871	**0.00653**	0.242	0.633
IL16					0.0303	0.117	0.384	0.11	0.0415
					0.929	0.731	0.348	0.748	0.904
IL17A						-0.339	**0.888**	0.089	-0.119

						0.307	**0.00325**	0.795	0.728
IL1a							0.00686	-0.556	-0.225
							0.987	0.0755	0.505
IL5								0.198	0.116
								0.638	0.785
IL7									**0.835**
									**0.00138**

	IFNg	IL10	IL12-70	IL13	IL1b	IL2	IL4	IL6	IL8
CRP	-0.00748	0.0419	-0.297	-0.227	1	0.718	-0.321	0.575	-0.177
	0.983	0.903	0.438	0.502	--	0.0692	0.367	0.0643	0.602
ICAM-1	**0.645**	0.0114	-0.071	-0.516	-1	0.312	**-0.73**	0.595	0.0111
	**0.032**	0.973	0.856	0.104	--	0.496	**0.0165**	0.0535	0.974
SAA	0.0989	0.166	-0.276	-0.199	1	0.63	-0.278	**0.662**	-0.00426
	0.772	0.625	0.472	0.558	--	0.13	0.437	**0.0265**	0.99
VCAM	0.327	0.361	-0.0829	0.0944	-1	0.18	-0.422	0.439	0.251
	0.327	0.276	0.832	0.782	--	0.699	0.224	0.177	0.456
FLT-1	0.0301	0.2	-0.513	0.16	1	0.715	-0.231	0.338	-0.315
	0.93	0.556	0.158	0.638	--	0.0708	0.521	0.309	0.346

PIGF	0.328	0.0784	-0.587	-0.132	1	0.586	-0.299	0.521	-0.031
	0.324	0.819	0.0965	0.7	--	0.167	0.401	0.1	0.928
Tie-2	0.574	0.212	0.00918	-0.219	-1	0.6	-0.143	0.594	0.0803
	0.0647	0.531	0.981	0.518	--	0.154	0.693	0.0541	0.814
VEGF-C	-0.365	0.335	0.331	0.288	1	-0.452	-0.248	-0.475	0.493
	0.269	0.313	0.384	0.391	--	0.309	0.49	0.14	0.124
VEGF-D	0.0232	-0.233	-0.212	-0.243	1	0.265	-0.214	0.306	0.00844
	0.946	0.491	0.585	0.471	--	0.566	0.553	0.361	0.98
bFGF	-0.433	0.152	-0.479	0.156	1	0.402	-0.0849	-0.38	0.153
	0.184	0.654	0.192	0.647	--	0.371	0.816	0.25	0.653
Eotaxin	-0.269	-0.218	-0.192	-0.122	0	0.579	-0.0503	-0.49	0.162
	0.52	0.605	0.679	0.773	--	0.306	0.906	0.218	0.702
eotaxin-3	-0.0417	-0.0305	-0.0773	0.239	-1	-0.18	-0.09	-0.438	0.147
	0.915	0.938	0.856	0.535	--	0.733	0.818	0.239	0.705
IP-10	-0.142	0.58	-0.0623	0.591	-1	-0.284	0.129	-0.293	0.49
	0.678	0.0612	0.874	0.0555	--	0.537	0.722	0.382	0.126
MCP-1	-0.347	0.414	**0.712**	0.454	-1	-0.58	-0.0544	-0.458	0.573
	0.295	0.206	**0.0314**	0.16	--	0.173	0.881	0.156	0.0656

MCP-4	-0.22	0.356	-0.338	0.228	0	0.654	-0.143	-0.223	0.36
	0.6	0.386	0.458	0.588	--	0.231	0.736	0.595	0.38
MDC	-0.507	0.194	0.398	0.591	0	-0.109	-0.419	-0.243	0.25
	0.134	0.591	0.328	0.0717	--	0.837	0.261	0.499	0.486
MIP-1a	0.572	0.585	-0.657	0.511	0	0.594	0.0449	0.381	0.298
	0.138	0.127	0.109	0.195	--	0.291	0.916	0.352	0.474
MIP-1b	-0.01	0.217	0.0489	0.717	-1	-0.691	0.419	-0.598	0.353
	0.98	0.575	0.908	0.0297	--	0.129	0.261	0.0887	0.351
TARC	-0.248	0.339	0.0826	0.342	-1	-0.742	-0.128	-0.401	0.533
	0.49	0.339	0.833	0.333	--	0.0912	0.725	0.25	0.113
GM-CSF	-0.367	-0.332	-0.365	-0.541	1	0.648	-0.147	0.0674	**-0.816**
	0.331	0.382	0.374	0.133	--	0.237	0.706	0.863	**0.00733**
IL12-23	0.113	0.327	-0.406	0.101	1	0.762	-0.294	0.5	0.0995
	0.741	0.327	0.279	0.767	--	0.0463	0.409	0.118	0.771
IL15	0.118	0.159	-0.139	-0.185	1	0.604	-0.246	0.406	0.0747
	0.729	0.641	0.722	0.586	--	0.151	0.492	0.215	0.827
IL16	0.445	0.45	0.281	-0.148	-1	0.0478	-0.209	0.552	**0.64**
	0.17	0.165	0.464	0.663	--	0.919	0.561	0.0783	**0.034**
IL17A	-0.182	-0.175	-0.405	-0.189	1	0.57	-0.241	0.136	-0.122

	0.592	0.606	0.279	0.578	--	0.181	0.503	0.691	0.72
IL1a	0.345	-0.0362	-0.137	-0.233	-1	0.537	-0.325	**0.619**	-0.443
	0.299	0.916	0.726	0.491	--	0.214	0.36	**0.0422**	0.173
IL5	-0.15	0.279	-0.214	0.0367	0	0.931	-0.354	0.509	-0.00533
	0.722	0.504	0.684	0.931	--	0.0694	0.436	0.197	0.99
IL7	-0.314	0.439	0.356	0.38	1	-0.287	0.0624	-0.442	0.552
	0.347	0.176	0.347	0.25	--	0.533	0.864	0.174	0.0782
TNFb	-0.41	0.514	0.29	0.656	-1	-0.138	-0.0248	-0.419	0.485
	0.21	0.106	0.448	0.0285	--	0.768	0.946	0.199	0.13
IFNg		0.0889	-0.331	-0.365	-1	-0.00082	0.334	**0.612**	0.219
		0.795	0.384	0.27	--	0.999	0.346	**0.0452**	0.517
IL10			-0.117	0.57	-1	0.0635	0.209	0.299	0.662
			0.764	0.0671	--	0.892	0.562	0.372	0.0266
IL12-70				-0.116	-1	-0.545	-0.225	-0.361	0.286
				0.767	--	0.263	0.56	0.34	0.456
IL13					-1	-0.188	**0.635**	-0.325	0.41
					--	0.686	**0.0487**	0.33	0.21
IL1b						1	-1	-1	-1

						--	--	--	--
						2	2	2	2
IL2							-0.579	0.645	-0.488
							0.229	0.118	0.267
IL4								-0.239	0.212
								0.505	0.557
IL6									-0.0466
									0.892

	TNFa	VEGF-A	
CRP	0.223	-0.212	
	0.511	0.531	
ICAM-1	**0.63**	0.0861	
	**0.0377**	0.801	
SAA	0.337	-0.221	
	0.311	0.514	
VCAM	0.683	-0.205	
	0.0206	0.545	

FLT-1	0.527	-0.372	
	0.0959	0.259	
PIGF	0.541	0.323	
	0.0857	0.333	
Tie-2	**0.617**	-0.316	
	**0.0432**	0.343	
VEGF-C	-0.409	**0.638**	
	0.212	**0.0347**	
VEGF-D	0.088	0.129	
	0.797	0.706	
bFGF	-0.244	**0.748**	
	0.47	**0.00807**	
Eotaxin	-0.183	**0.868**	
	0.664	**0.00523**	

eotaxin-3	0.163	0.362	
	0.675	0.339	
IP-10	-0.0743	0.314	
	0.828	0.347	
MCP-1	-0.489	0.219	
	0.127	0.517	
MCP-4	-0.00498	**0.727**	
	0.991	**0.0409**	
MDC	-0.129	0.242	
	0.722	0.501	
MIP-1a	**0.778**	-0.0409	
	**0.023**	0.923	
MIP-1b	-0.0837	0.135	
	0.831	0.73	

TARC	-0.177	**0.76**	
	0.625	**0.0108**	
GM-CSF	-0.126	-0.356	
	0.747	0.347	
IL12-23	0.426	-0.154	
	0.192	0.652	
IL15	0.076	0.00656	
	0.824	0.985	
IL16	0.389	0.177	
	0.237	0.602	
IL17A	-0.00628	0.166	
	0.985	0.626	
IL1a	0.55	-0.248	
	0.0799	0.463	
IL5	0.27	-0.054	
	0.519	0.899	
IL7	-0.469	0.358	
	0.146	0.28	

TNFb	-0.28	0.429	
	0.404	0.188	
IFNg	**0.672**	-0.339	
	**0.0234**	0.308	
IL10	0.392	-0.0614	
	0.233	0.858	
IL12-70	-0.578	0.162	
	0.103	0.677	
IL13	-0.0289	0.00711	
	0.933	0.983	
IL1b	-1	-1	
	--	--	
	2	2	
IL2	0.47	-0.339	
	0.287	0.456	
IL4	-0.0487	-0.308	
	0.894	0.387	
IL6	**0.801**	-0.428	
	**0.00305**	0.189	

IL8	0.129	0.258	
	0.705	0.444	
TNFa		-0.276	
		0.412	

Pearson Correlation of HP positive group
Upper number is Correlation Coefficient. Lower number is P-value

	ICAM-1	SAA	VCAM	FLT-1	PIGF	Tie-2	VEGF-C	VEGF-D
CRP	0.238	**0.897**	0.0577	0.145	0.04	0.136	-0.0395	0.243
	0.0562	**5.73E-24**	0.648	0.248	0.752	0.279	0.755	0.0513
ICAM-1		0.151	**0.544**	0.366	0.191	0.178	-0.0874	0.331
		0.229	**2.87E-06**	0.00275	0.127	0.157	0.489	0.00712
SAA			0.124	0.0751	-0.00166	0.173	0.0136	0.281
			0.324	0.552	0.99	0.168	0.914	0.0232
VCAM				0.0725	0.333	0.1	-0.141	0.26
				0.566	0.00669	0.426	0.262	0.0363
FLT-1					0.0037	0.103	0.181	0.126
					0.977	0.412	0.149	0.318
PIGF						0.0802	-0.143	0.0831
						0.526	0.257	0.511
Tie-2							-0.0833	0.305
							0.509	0.0136
VEGF-C								0.131
								0.299

VEGF-D								
bFGF								
Eotaxin								
eotaxin-3								
IP-10								
MCP-1								
MCP-4								
MDC								
MIP-1a								
MIP-1b								
TARC								
GM-CSF								
IL12-23								

IL15								
IL16								
IL17A								
IL1a								
IL5								
IL7								
TNFb								
IFNg								
IL10								
IL12-70								
IL13								
IL1b								
IL2								
IL4								

IL6								
IL8								
TNFa								
VEGF-A								
	bFGF	Eotaxin	eotaxin-3	IP-10	MCP-1	MCP-4	MDC	MIP-1a
CRP	-0.0254	-0.0528	-0.0092	-0.0604	-0.0959	-0.0817	-0.0129	0.00317
	0.841	0.688	0.945	0.632	0.447	0.546	0.925	0.982
ICAM-1	-0.0413	-0.101	-0.0095	0.215	-0.0466	0.0173	0.0747	0.252
	0.744	0.441	0.943	0.0852	0.712	0.898	0.584	0.0688
SAA	-0.0153	0.0174	0.0112	-0.0637	-0.0424	-0.00335	-0.101	0.000813
	0.904	0.895	0.933	0.614	0.737	0.98	0.459	0.995
VCAM	0.114	-0.109	-0.0839	0.221	-0.049	-0.065	-0.149	0.379
	0.367	0.406	0.527	0.0766	0.698	0.631	0.274	0.00515
FLT-1	0.0499	0.209	0.254	0.295	0.192	0.19	0.176	0.321
	0.693	0.108	0.0518	0.0172	0.125	0.156	0.194	0.0193
PIGF	0.0607	0.00187	0.242	0.0112	-0.035	0.147	-0.0179	0.0418
	0.631	0.989	0.0649	0.929	0.782	0.276	0.896	0.766

Tie-2	0.142	0.15	0.114	-0.195	0.0856	-0.165	0.0135	0.248
	0.26	0.253	0.391	0.12	0.498	0.221	0.921	0.0733
VEGF-C	0.0317	0.442	0.296	0.0622	**0.703**	**0.683**	0.346	0.0653
	0.802	0.0004	0.023	0.623	**6.91E-11**	**4.7E-09**	0.00904	0.642
VEGF-D	0.00708	0.203	0.103	0.145	0.137	0.0695	0.104	0.396
	0.955	0.119	0.439	0.25	0.278	0.607	0.445	0.00332
bFGF		0.0168	-0.0014	0.0574	0.00201	0.00795	-0.0413	0.171
		0.899	0.992	0.65	0.987	0.953	0.763	0.222
Eotaxin			**0.661**	0.33	**0.712**	**0.624**	0.479	0.373
			**1.69E-08**	0.00995	**1.76E-10**	**2.79E-07**	0.000188	0.00592
eotaxin-3				0.235	**0.525**	0.303	0.149	0.212
				0.0727	**0.00002**	0.0246	0.272	0.131
IP-10					0.276	0.122	0.307	0.283
					0.026	0.367	0.0216	0.0402
MCP-1						**0.663**	0.234	0.245
						**1.88E-08**	0.082	0.0769

MCP-4							0.365	0.188
							0.0066	0.186
MDC								0.198
								0.159
MIP-1a								
MIP-1b								
TARC								
GM-CSF								
IL12-23								
IL15								
IL16								
IL17A								
IL1a								
IL5								
IL7								

TNFb								
IFNg								
IL10								
IL12-70								
IL13								
IL1b								
IL2								
IL4								
IL6								
IL8								
TNFa								
VEGF-A								
	MIP-1b	TARC	GM-CSF	IL12-23	IL15	IL16	IL17A	IL1a
CRP	-0.0474	-0.108	-0.135	-0.171	-0.0722	0.148	0.0996	-0.149
	0.729	0.407	0.325	0.172	0.568	0.239	0.43	0.236

ICAM-1	-0.0394	0.0591	0.123	0.297	0.0246	0.348	0.187	0.038
	0.773	0.651	0.371	0.0163	0.846	0.00452	0.135	0.764
SAA	-0.0221	-0.08	-0.149	-0.131	0.0747	0.0813	0.0557	-0.158
	0.872	0.54	0.278	0.299	0.554	0.519	0.659	0.208
VCAM	-0.124	-0.113	0.248	0.452	0.113	0.365	0.108	0.146
	0.363	0.387	0.0676	0.000159	0.369	0.00283	0.394	0.246
FLT-1	0.0676	0.0892	0.241	-0.00502	0.0679	0.161	0.146	-0.174
	0.621	0.494	0.0764	0.968	0.591	0.2	0.244	0.166
PIGF	-0.0398	0.00309	-0.0765	0.153	0.0505	0.197	-0.0667	-0.0362
	0.771	0.981	0.579	0.223	0.69	0.115	0.598	0.774
Tie-2	0.0847	0.102	-0.0419	-0.186	0.0299	0.075	-0.186	-0.141
	0.535	0.433	0.761	0.138	0.813	0.553	0.138	0.261
VEGF-C	0.0809	0.492	0.275	0.00551	0.296	-0.0652	0.0737	-0.195
	0.553	5.56E-05	0.0421	0.965	0.0167	0.606	0.56	0.119
VEGF-D	0.0754	0.152	0.12	-0.0287	0.304	0.0356	-0.026	-0.0896
	0.581	0.242	0.382	0.821	0.014	0.778	0.837	0.478
bFGF	0.0302	0.0704	0.0279	0.132	0.121	0.17	-0.0475	-0.0712
	0.825	0.59	0.84	0.295	0.337	0.177	0.707	0.573

Eotaxin	0.315	0.462	0.0287	-0.0419	0.531	-0.0219	-0.0405	-0.0918
	0.0179	0.000293	0.843	0.75	1.27E-05	0.868	0.759	0.485
eotaxin-3	0.105	0.322	-0.0407	-0.063	0.58	-0.134	0.0772	-0.138
	0.442	0.0166	0.779	0.635	1.51E-06	0.311	0.561	0.296
IP-10	0.0254	0.149	0.158	0.458	0.159	0.213	0.258	-0.101
	0.852	0.253	0.249	0.000125	0.207	0.0881	0.0377	0.425
MCP-1	0.101	0.269	0.232	-0.00141	0.47	-0.127	0.0157	0.0128
	0.459	0.0361	0.0888	0.991	7.74E-05	0.313	0.902	0.919
MCP-4	0.0908	0.367	0.0828	0.0056	0.25	-0.0687	-0.0107	0.169
	0.514	0.00539	0.58	0.967	0.0608	0.612	0.937	0.208
MDC	0.147	**0.676**	0.18	0.0498	-0.0114	0.0525	0.0393	-0.215
	0.279	**1.47E-08**	0.227	0.716	0.933	0.701	0.774	0.112
MIP-1a	0.109	0.0922	**0.581**	0.0566	0.346	0.0504	0.0164	0.193
	0.443	0.511	**3.57E-05**	0.687	0.0111	0.72	0.907	0.167
MIP-1b		0.189	0.0297	0.0357	0.192	-0.0179	-0.00347	-0.0876
		0.168	0.843	0.794	0.157	0.896	0.98	0.521
TARC			0.035	0.0418	0.0795	0.156	-0.0186	-0.171
			0.807	0.749	0.542	0.231	0.887	0.187

GM-CSF				0.394	0.212	0.0616	0.257	0.121
				0.00295	0.121	0.655	0.0577	0.379
IL12-23					0.0775	0.364	0.516	-0.0552
					0.539	0.0029	1.11E-05	0.662
IL15						0.0179	0.00524	-0.0339
						0.888	0.967	0.788
IL16							-0.0191	-0.141
							0.88	0.261
IL17A								-0.0865
								0.493
IL1a								
IL5								
IL7								
TNFb								
IFNg								
IL10								
IL12-70								

IL13								
IL1b								
IL2								
IL4								
IL6								
IL8								
TNFa								
VEGF-A								
	IL5	IL7	TNFb	IFNg	IL10	IL12-70	IL13	IL1b
CRP	0.217	-0.117	-0.0373	-0.0528	-0.0832	-0.0507	-0.0422	**0.968**
	0.101	0.354	0.768	0.676	0.51	0.7	0.771	**4.41E-06**
ICAM-1	0.148	-0.164	0.111	0.153	0.131	0.21	0.036	**0.662**
	0.268	0.193	0.379	0.225	0.298	0.108	0.804	**0.0371**
SAA	0.244	-0.0559	-0.031	-0.00501	-0.104	-0.0797	-0.0619	**0.858**
	0.0653	0.658	0.807	0.968	0.411	0.545	0.67	**0.0015**
VCAM	0.28	-0.168	0.267	0.178	0.281	0.201	0.0751	-0.061

	0.033	0.18	0.0315	0.156	0.0236	0.124	0.604	0.867
FLT-1	0.123	0.205	0.201	0.272	0.123	0.376	0.189	-0.0833
	0.359	0.102	0.109	0.0286	0.327	0.00306	0.189	0.819
PIGF	0.137	-0.158	-0.0719	0.00187	-0.0827	-0.172	-0.0701	**0.794**
	0.303	0.209	0.569	0.988	0.513	0.19	0.629	**0.00613**
Tie-2	-0.189	-0.018	-0.175	-0.162	-0.195	-0.0243	-0.0737	0.388
	0.155	0.887	0.164	0.197	0.12	0.854	0.611	0.268
VEGF-C	0.0977	0.565	0.227	-0.0645	-0.0739	0.174	0.108	0.18
	0.466	9.67E-07	0.0696	0.61	0.559	0.184	0.455	0.619
VEGF-D	0.269	-0.0455	0.097	0.203	0.0472	0.236	0.11	0.521
	0.0411	0.719	0.442	0.105	0.709	0.0698	0.447	0.122
bFGF	-0.0859	0.442	-0.121	-0.0868	-0.0174	-0.0639	-0.0416	0.195
	0.521	0.000225	0.338	0.492	0.89	0.628	0.774	0.59
Eotaxin	0.138	0.339	0.0798	0.023	-0.0977	0.0725	-0.151	-0.0771
	0.32	0.00801	0.544	0.861	0.458	0.599	0.321	0.832
eotaxin-3	0.103	0.239	0.0342	0.156	-0.071	0.0761	-0.00269	-0.105
	0.462	0.0679	0.797	0.238	0.593	0.585	0.986	0.773

IP-10	0.318	0.27	0.178	0.161	0.102	0.266	0.0386	-0.0898
	0.015	0.0295	0.156	0.201	0.421	0.0398	0.79	0.805
MCP-1	0.138	0.392	0.284	-0.0287	-0.082	0.235	0.184	-0.00114
	0.3	0.00124	0.0219	0.82	0.516	0.0708	0.201	0.998
MCP-4	0.162	0.453	0.167	0.00426	0.0386	0.0762	0.025	0.169
	0.248	0.000401	0.215	0.975	0.776	0.591	0.872	0.688
MDC	0.264	0.155	0.165	-0.061	0.2	0.23	0.0965	0.0785
	0.0616	0.253	0.226	0.655	0.139	0.105	0.543	0.853
MIP-1a	**0.549**	0.0644	**0.502**	**0.509**	0.469	**0.604**	0.347	-0.0292
	**6.35E-05**	0.647	**0.000127**	**9.95E-05**	0.000396	**5.38E-06**	0.0304	0.951
MIP-1b	-0.0811	0.0744	-0.0256	0.285	0.0444	-0.0383	-0.0747	0.088
	0.572	0.586	0.852	0.0333	0.745	0.789	0.638	0.836
TARC	-0.00287	0.262	0.00384	-0.115	0.0769	0.201	0.136	-0.0805
	0.983	0.0411	0.977	0.377	0.556	0.137	0.366	0.864
GM-CSF	**0.542**	0.139	**0.836**	0.434	**0.511**	**0.774**	**0.567**	-0.304
	**6.93E-05**	0.311	**2.10E-15**	0.000942	**6.64E-05**	**1.78E-11**	**4.85E-05**	0.393
IL12-23	0.112	0.212	0.266	0.251	0.376	0.131	0.103	-0.483

	0.405	0.0901	0.0322	0.044	0.00203	0.32	0.475	0.158
IL15	0.203	0.137	0.196	0.404	0.102	0.163	0.00201	-0.148
	0.126	0.278	0.118	0.000851	0.419	0.213	0.989	0.683
IL16	-0.06	0.00521	-0.142	-0.0581	0.0637	-0.108	-0.208	0.456
	0.655	0.967	0.259	0.646	0.614	0.411	0.147	0.185
IL17A	0.215	0.292	0.335	0.156	0.207	0.186	0.244	-0.477
	0.105	0.0182	0.00646	0.216	0.0987	0.155	0.0878	0.164
IL1a	0.168	-0.221	0.375	0.0837	0.344	0.284	0.466	-0.328
	0.208	0.0774	0.00212	0.508	0.00508	0.028	0.000655	0.354
IL5		-0.00078	0.732	0.346	0.345	0.616	0.423	-0.213
		0.995	6.60E-11	0.00776	0.00802	8.98E-07	0.00378	0.583
IL7			0.033	-0.0377	0.0118	0.0352	-0.0232	-0.173
			0.794	0.765	0.926	0.789	0.873	0.634
TNFb				0.442	**0.528**	**0.847**	**0.702**	-0.386
				0.000228	**6.33E-06**	**1.57E-17**	**1.37E-08**	0.271
IFNg					**0.629**	**0.504**	0.378	-0.197
					**2.02E-08**	**4.07E-05**	0.00686	0.585

IL10						**0.567**	**0.513**	-0.213
						**2.31E-06**	**0.000142**	0.554
IL12-70							**0.716**	-0.229
							**1.51E-08**	0.554
IL13								-0.204
								0.598

	IL2	IL4	IL6	IL8	TNFa	VEGF-A
CRP	-0.158	-0.057	**0.729**	-0.0719	-0.0966	-0.125
	0.389	0.679	**5.52E-12**	0.569	0.444	0.321
ICAM-1	-0.00463	0.0307	0.312	0.354	0.428	-0.0348
	0.98	0.824	0.0114	0.00386	0.000374	0.783
SAA	-0.156	-0.0579	**0.798**	-0.102	-0.0957	-0.077
	0.395	0.675	**1.66E-15**	0.419	0.448	0.542
VCAM	-0.0262	0.0794	0.324	0.093	0.563	-0.117
	0.887	0.564	0.00839	0.461	1.05E-06	0.355
FLT-1	0.268	0.174	0.3	0.368	0.285	0.0199
	0.139	0.204	0.0151	0.00257	0.0212	0.875
PIGF	-0.151	-0.19	0.179	0.206	0.1	-0.0973
	0.408	0.165	0.153	0.0996	0.427	0.441

Tie-2	0.102	-0.0935	0.214	0.0396	-0.0524	0.0106
	0.58	0.497	0.0868	0.754	0.678	0.933
VEGF-C	0.0895	**0.517**	-0.104	0.302	0.0166	**0.68**
	0.626	**5.35E-05**	0.412	0.0146	0.896	**4.51E-10**
VEGF-D	-0.0571	0.0837	0.298	0.139	0.108	0.057
	0.756	0.543	0.0159	0.268	0.391	0.652
bFGF	0.173	-0.0837	-0.0208	-0.0529	0.232	0.218
	0.344	0.544	0.869	0.676	0.0632	0.0805
Eotaxin	-0.161	0.184	0.00817	0.369	-0.0679	**0.572**
	0.404	0.201	0.951	0.00371	0.606	**1.85E-06**
eotaxin-3	0.279	0.0683	0.0653	**0.509**	-0.0502	0.493
	0.142	0.641	0.623	**0.000039**	0.706	7.19E-05
IP-10	0.166	0.159	0.0531	0.159	0.366	0.088
	0.365	0.247	0.675	0.206	0.0027	0.486
MCP-1	0.342	0.442	0.019	0.421	0.0352	**0.53**
	0.0553	0.00072	0.881	0.000485	0.781	**5.55E-06**

MCP-4	-0.174	0.268	-0.00532	0.298	0.067	0.472
	0.405	0.0688	0.969	0.0243	0.62	0.000212
MDC	-0.0194	0.403	-0.0919	0.0768	0.00586	0.442
	0.925	0.00496	0.5	0.574	0.966	0.000641
MIP-1a	0.243	0.36	0.248	0.185	0.434	0.213
	0.252	0.0164	0.0739	0.184	0.00117	0.125
MIP-1b	0.0889	-0.0204	-0.0634	0.0148	-0.0832	0.142
	0.666	0.892	0.642	0.914	0.542	0.298
TARC	**0.514**	0.421	-0.103	0.0765	-0.01	**0.689**
	**0.0051**	0.00188	0.428	0.558	0.939	**8.21E-10**
GM-CSF	0.4	**0.796**	0.106	0.237	**0.554**	0.263
	0.0388	**2.30E-11**	0.44	0.0808	**1.14E-05**	0.052
IL12-23	0.115	0.186	0.000679	0.204	**0.703**	0.0135
	0.532	0.174	0.996	0.104	**6.78E-11**	0.915
IL15	-0.126	0.197	0.122	0.451	0.124	0.41
	0.492	0.149	0.331	0.000166	0.325	0.000694
IL16	-0.125	-0.0804	0.221	-0.129	0.395	0.00546
	0.494	0.559	0.0771	0.304	0.00113	0.966

IL17A	0.143	0.345	0.15	0.23	0.307	-0.00401
	0.434	0.00985	0.233	0.0648	0.0129	0.975
IL1a	0.404	0.137	-0.0169	-0.0862	0.151	-0.0169
	0.022	0.32	0.894	0.495	0.23	0.894
IL5	0.00614	**0.558**	0.395	0.105	0.295	0.0951
	0.977	**3.16E-05**	0.00213	0.433	0.0248	0.478
IL7	0.0574	0.226	-0.114	0.204	0.123	0.452
	0.755	0.0971	0.366	0.103	0.331	0.000159
TNFb	0.441	**0.805**	0.235	0.233	0.446	0.166
	0.0115	**1.27E-13**	0.0598	0.062	0.000198	0.185
IFNg	0.189	0.183	0.186	0.263	0.429	-0.0317
	0.299	0.18	0.138	0.0344	0.000359	0.802
IL10	0.197	0.287	0.126	0.0873	**0.555**	0.0407
	0.279	0.0334	0.319	0.489	**1.62E-06**	0.748
IL12-70	0.445	**0.692**	0.229	0.289	0.432	0.176
	0.0122	**1.31E-08**	0.0788	0.025	0.00057	0.179
IL13	**0.591**	**0.634**	0.237	0.0938	0.482	0.132

	**0.00187**	**2.89E-06**	0.0974	0.517	0.00039	0.361
IL1b	-0.345	-0.457	0.0649	-0.219	0.0596	-0.082
	0.403	0.302	0.859	0.543	0.87	0.822
IL2		**0.575**	0.162	0.0619	0.319	0.034
		**0.00111**	0.375	0.737	0.0747	0.854
IL4			0.112	0.194	0.377	0.516
			0.415	0.157	0.00459	5.53E-05
IL6				0.0877	0.211	-0.155
				0.487	0.0908	0.218
IL8					0.232	0.285
					0.0632	0.0216
TNFa						0.0466
						0.713
